# Chronic graft-versus-host disease detected by tissue-specific cell-free DNA methylation biomarkers

**DOI:** 10.1172/JCI163541

**Published:** 2024-01-16

**Authors:** Batia Avni, Daniel Neiman, Elior Shaked, Ofer Gal-Rosenberg, Sigal Grisariu, Mona Kuzli, Ilai Avni, Andrea Fracchia, Polina Stepensky, Tsila Zuckerman, Ahinoam Lev-Sagie, Ilana Fox-Fisher, Sheina Piyanzin, Joshua Moss, Seth J. Salpeter, Benjamin Glaser, Ruth Shemer, Yuval Dor

**Affiliations:** 1 Bone Marrow Transplantation and Cancer Immunotherapy Department, Hadassah University Medical Center and Faculty of Medicine, the Hebrew University, Jerusalem, Israel.; 2 Department of Developmental Biology and Cancer Research, Institute for Medical Research Israel-Canada, the Hebrew University-Hadassah Medical School, Jerusalem, Israel.; 3 Faculty of Data and Decision Sciences, Institute of Technology — Technion, Haifa, Israel.; 4 Hematology Institute and Bone Marrow Transplantation, Rambam Health Care Campus, Haifa, Israel.; 5 Department of Obstetrics and Gynecology, Hadassah University Medical Center and Faculty of Medicine, the Hebrew University, Jerusalem, Israel.; 6 Endocrinology and Metabolism Service, Hadassah University Medical Center and Faculty of Medicine, the Hebrew University, Jerusalem, Israel.

**Keywords:** Transplantation, Stem cell transplantation

## Abstract

**BACKGROUND.** Accurate detection of graft-versus-host disease (GVHD) is a major challenge in the management of patients undergoing hematopoietic stem cell transplantation (HCT). Here, we demonstrated the use of circulating cell-free DNA (cfDNA) for detection of tissue turnover and chronic GVHD (cGVHD) in specific organs.

**METHODS.** We established a cocktail of tissue-specific DNA methylation markers and used it to determine the concentration of cfDNA molecules derived from the liver, skin, lungs, colon, and specific immune cells in 101 patients undergoing HCT.

**RESULTS.** Patients with active cGVHD showed elevated concentrations of cfDNA, as well as tissue-specific methylation markers that agreed with clinical scores. Strikingly, transplanted patients with no clinical symptoms had abnormally high levels of tissue-specific markers, suggesting hidden tissue turnover even in the absence of evident clinical pathology. An integrative model taking into account total cfDNA concentration, monocyte/macrophage cfDNA levels and alanine transaminase was able to correctly identify GVHD with a specificity of 86% and precision of 89% (AUC of 0.8).

**CONCLUSION.** cfDNA markers can be used for the detection of cGVHD, opening a window into underlying tissue dynamics in patients that receive allogeneic stem cell transplants.

**FUNDING.** This work was supported by grants from the Ernest and Bonnie Beutler Research Program of Excellence in Genomic Medicine, The Israel Science Foundation, the Waldholtz/Pakula family, the Robert M. and Marilyn Sternberg Family Charitable Foundation and the Helmsley Charitable Trust (to YD).

## Abstract 2

Tissue-specific DNA methylation markers allow the monitoring of tissue damage in chronic graft-versus-host disease via circulating cell-free DNA.

## Graphical abstract

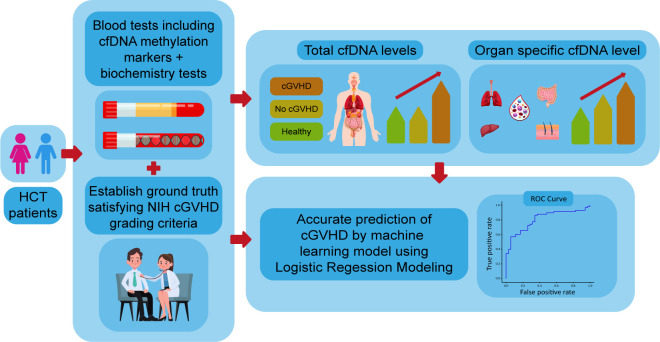

## Introduction

Hematopoietic stem cell transplantation (HCT) is an essential and often the sole curative treatment strategy for high risk hematologic malignancies (1). Graft-versus-host disease (GVHD), the foremost complication of allogeneic HCT, is a major limitation of this procedure, accounting for deleterious effects on quality of life and increased mortality from HCT (2, 3). Current diagnosis of acute GVHD (aGVHD) and chronic GVHD (cGVHD) in patents who have undergone a bone marrow transplant is based on inaccurate, operator-dependent clinical markers, and less often on biopsies. These methods are time consuming, costly, invasive, and yield late-stage diagnoses that negatively affect morbidity and mortality. In addition, current practice lacks accurate biomarkers for prediction of disease occurrence, identification of disease onset, prediction of disease response to treatment, and accurate assessment of the actual response to treatment (4). Multiple prognostic and diagnostic biomarkers for cGVHD have been proposed, including IL2Rα, aminopeptidase N (CD13), IL4, IL6, TNFα, ST2, OPN, chemokine ligands such as CXCL9, CXCL10, and CXCL11 (5–14), cellular biomarkers including immune cells subpopulations (15–18), miRNA (19), and others. However, none of these biomarkers have been clinically validated. In addition, all these markers are indicative of immune system derangement, lacking information on the damaged tissue targeted by the alloimmune process. Thus, there is an unmet need for simple objective tools that can aid the treating physician in easier identification and scoring, and can assist with personalization of management in patients suffering from cGVHD.

Classic liquid biopsies analyze circulating cell-free DNA (cfDNA) via genetic variations or mutations in the DNA of a fetus, a tumor, or a transplanted solid organ. However, these approaches are blind to DNA released from cells with a normal genome, as would occur in organs damaged by pathologies such as GVHD. We and others have previously shown that tissue-specific DNA methylation patterns can provide powerful, universal biomarkers for detecting the tissue origins of cfDNA, reflective of elevated turnover or damage in specific organs and regardless of the underlying pathology (20–22). For example, we showed that genomic loci specifically unmethylated in lung epithelial cells or in hepatocytes can serve as cfDNA biomarkers to detect specific lung or liver injury (20–26).

The aim of this study was to establish a set of affordable, yet highly specific and sensitive methylation markers for cell types relevant to patients at risk for developing cGVHD, to examine their utility for detection of damage to specific organs in patients with clinically suspected cGVHD, and to create a cfDNA-based model that can assist the treating physician in surveillance and treatment decisions.

## Results

### DNA methylation markers for targeted assessment of cGVHD-relevant tissue damage.

**Figure 1:**
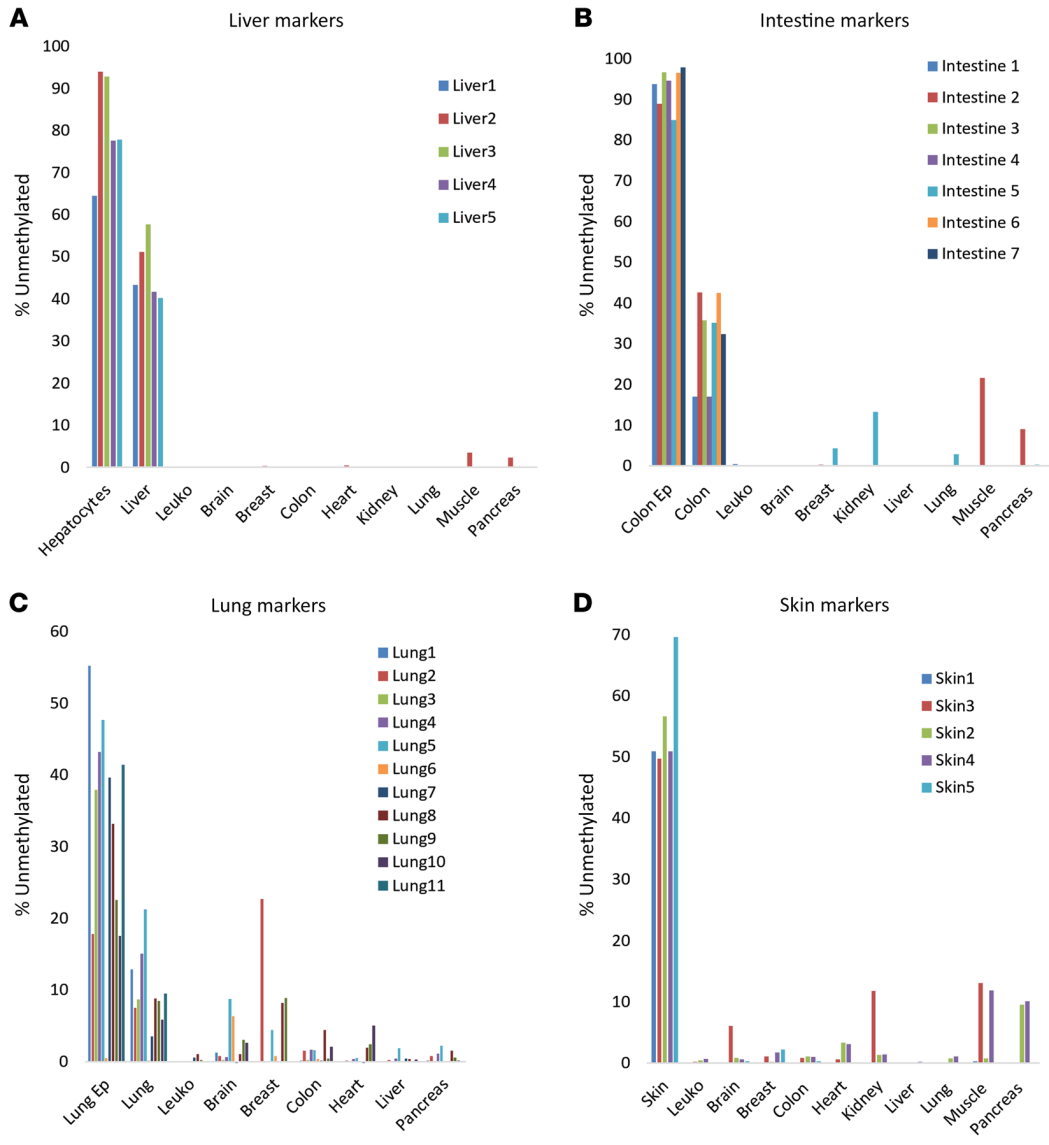
Characterization of methylation biomarkers. Tissue specificity of methylation markers for liver (**A**), intestinal epithelium (**B**), lung epithelium (**C**), and skin (**D**). Each color represents a different marker.

We compared publicly available methylomes of specific human tissues (21) and identified genomic loci containing CpG sites that are uniquely unmethylated in specific tissues or cell types, relevant to cGVHD. These included hepatocytes (5 markers), skin (5 markers), lung epithelial cells (10 markers), and intestinal epithelial cells (8 markers). We designed multiplex PCR cocktails to amplify all these loci from genomic DNA after bisulfite conversion and sequenced the products to determine the fraction of unmethylated DNA molecules present in the starting material. Figure 1 shows the fraction of methylation blocks from each marker locus that were unmethylated in the indicated samples. As we have shown previously, molecules containing multiple unmethylated CpG sites could be assigned with extreme specificity to a given tissue of origin. We also spiked genomic DNA from specific tissues into genomic DNA of leukocytes to determine assay sensitivity and linearity and found that as little as 0.5% of DNA from the target tissue could be robustly identified when present in a mixture (not shown). These findings establish a cocktail of DNA methylation markers that can be used to identify DNA derived from the liver, skin, lungs, and intestine with extreme specificity and sensitivity. We also used methylation markers specific to selected immune and inflammatory cell types: neutrophils, eosinophils, monocytes, B lymphocytes, and T lymphocytes (including CD8^^+^^ and regulatory T cells); all of which showed extreme specificity and sensitivity (27).

### Elevated cfDNA levels in patients undergoing HCT with and without clinical GVHD.

**Figure 2:**
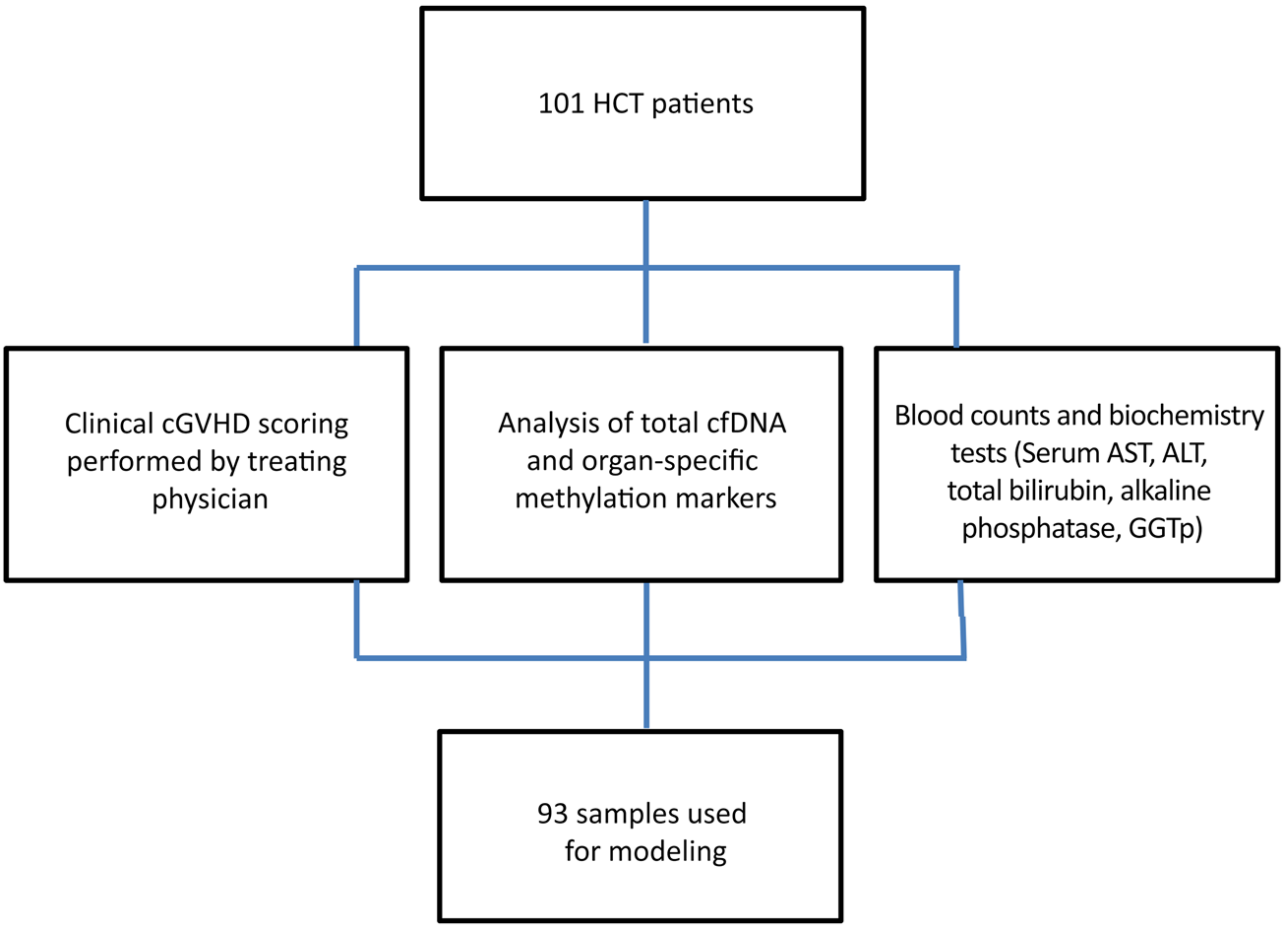
Experimental design. 101 plasma samples were collected from 101 individuals arriving for planned clinical follow up at the BMT day care unit. Upon each visit blood was drawn for regular blood tests and the patient underwent a full assessment by the treating physician, which included cGVHD grading according to the 2014 NIH criteria. 93 samples were employed for modeling, excluding those with missing data. cf, cell free; AST, aspartate transaminase; ALP, alkaline phosphatase; GGTp, γ glutamyl transpeptidase.

The overall scheme of the experiment is shown in Figure 2. We recruited a total of 101 patients who underwent HCT, obtained blood samples, and recorded clinical cGVHD scores as well as blood counts and standard blood biochemistry. We determined plasma cfDNA concentration and methylation patterns, compared findings to clinical and biochemistry data, and then developed and validated a model for inference of cGVHD based on cfDNA parameters combined with blood biochemistry markers (Figure 2). The characteristics of the 101 recruited patients and samples are detailed in Supplemental Tables 2–4 and in the Methods section; supplemental material available online with this article; https://doi.org/10.1172/JCI163541DS1

We compared total and tissue-specific cfDNA concentration in samples from healthy individuals (median age 37 years old (range 24–68), 58% women and 42% men), samples from patients who underwent HCT and had no evidence of cVHD from patients undergoing HCT defined by the treating physician as having clinically evident cGVHD. The NIH 2014 criteria were used for defining disease severity (mild, moderate, and severe) and organ scoring (range 0–3).

**Figure 3:**
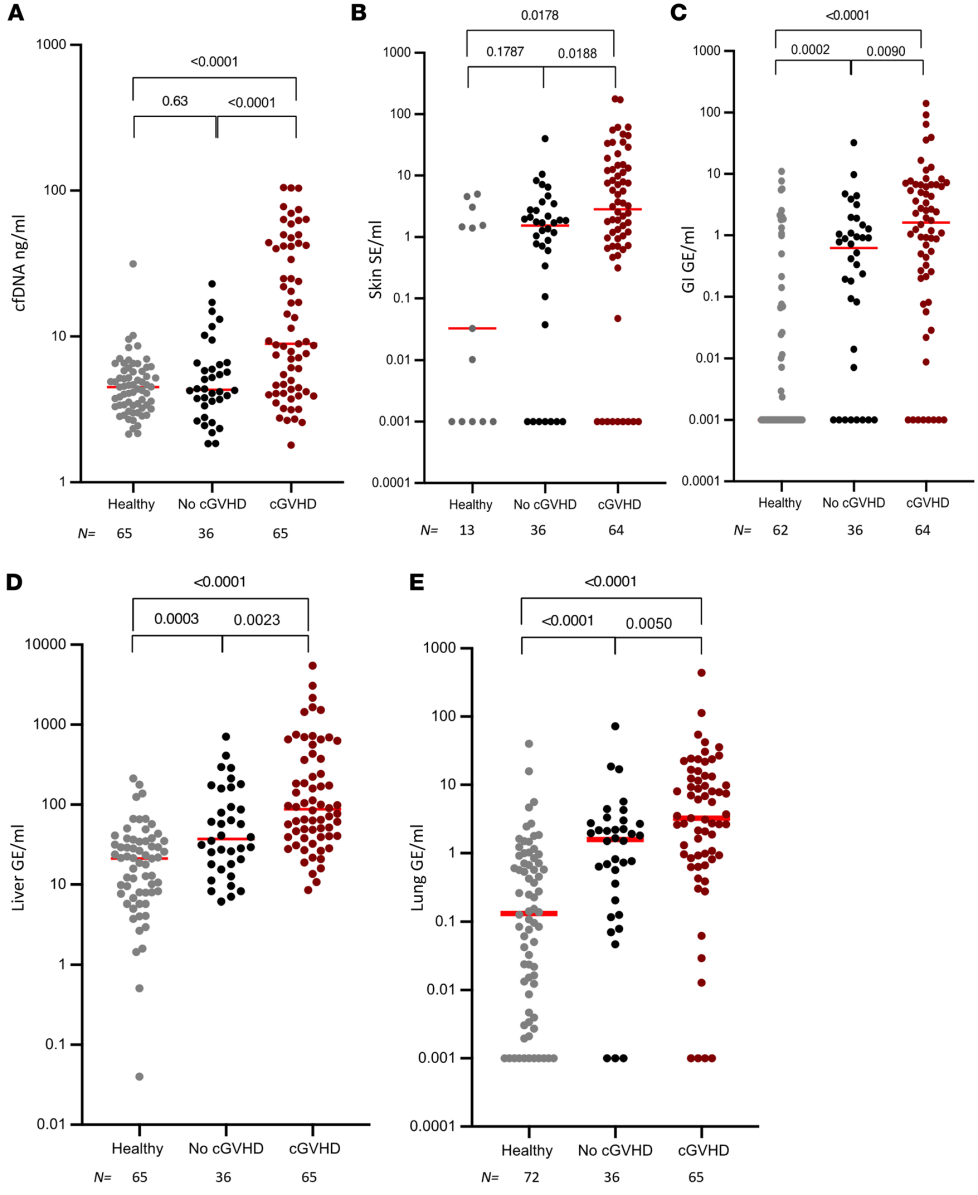
cfDNA levels correlate with clinical presence of cGVHD. (**A**) Level of total cfDNA in healthy volunteers and patients undergoing allogeneic HCT with and without clinical signs of cGVHD. (**B**–**E**) Tissue-specific cfDNA (genome equivalents per mL plasma) in healthy volunteers and patients undergoing allogeneic HCT with and without clinical signs of cGVHD, using average signals from methylation markers of skin (**B**), gastrointestinal tract (**C**), liver (**D**) and lung (**E**). Each dot represents 1 plasma sample. Statistical analysis was performed using nonparametric 2-tailed Mann-Whitney test. cfDNA ng/mL, total cell free DNA levels in ng/mL; GI, gastrointestinal.

Analyzing 101 samples from 101 patients; patients undergoing HCT with cGVHD (in any organ) had statistically significant higher concentrations of total cfDNA compared with patients undergoing HCT with no clinical evidence of cGVHD (*P <* 0.0001) (Figure 3A). Total cfDNA levels in patients undergoing HCT were similar to those in healthy controls (*P =* 0.63).

cfDNA signals from skin (*P =* 0.0188), intestine (*P =* 0.009), liver (*P =* 0.0023), and lungs (*P =* 0.0050) were also significantly higher in the group with clinically evident cGVHD compared with the group of patients who did not meet the NIH 2014 criteria for cGVHD (Figure 3, B–E). In addition, the concentration of cfDNA originating from GI, liver, and lung was significantly higher in patients who underwent HCT with no evidence of clinical GVHD compared with people in the healthy control group (*P =* 0.0002, *P =* 0.0003, and *P <* 0.0001, respectively) (Figure 3, C–E). Moreover, cfDNA originating from skin and liver significantly correlated with organ-specific clinical GVHD presence (score 0 versus score 1–3) (*P =* 0.0022, *P =* 0.0003, Supplemental Figure 1, A and C, respectively). Interestingly, patients undergoing HCT with and without lung cGVHD (score 0 versus 1–3) had significantly higher levels of lung cfDNA compared with healthy controls (*P <* 0.0001), but lung cfDNA did not correlate with the presence of clinical lung score (Supplemental Figure 1B).

**Figure 4:**
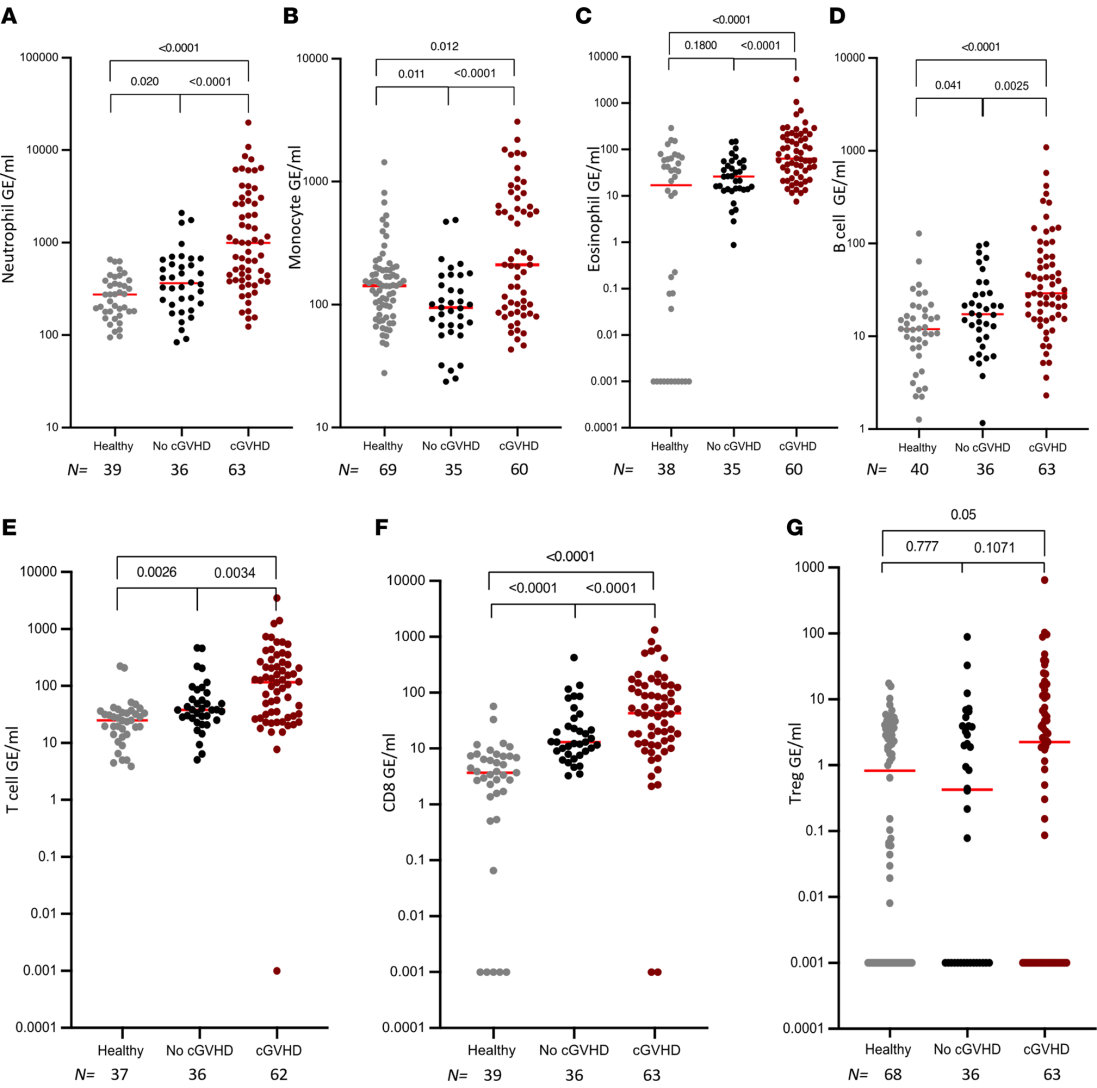
Immune-derived cfDNA levels correlate with clinical presence of chronic GVHD. Level of immune specific cfDNA in healthy volunteers and patients undergoing allogeneic HCT with and without clinical signs of cGVHD, using average signals from methylation markers of neutrophils (**A**), monocytes (**B**), eosinophils (**C**), B cells (**D**), T cells (**E**), CD8^+^ cells (**F**), and Tregs (**G**). Each dot represents 1 plasma sample. Statistical analysis was performed using nonparametric 2-tailed Mann-Whitney test.

Analysis of immune-derived cfDNA showed a significantly higher concentration of cfDNA originating from neutrophils, monocytes, eosinophils, and B and T lymphocytes in patients undergoing HCT diagnosed with clinical cGVHD compared with patients who were not diagnosed (Figure 4). cfDNA from neutrophils, T cells, and CD8^^+^^ T cells was elevated in patients undergoing HCT who have no clinical cGVHD compared with people in the healthy volunteer group (Figure 4, A, E, and F).

**Figure 5:**
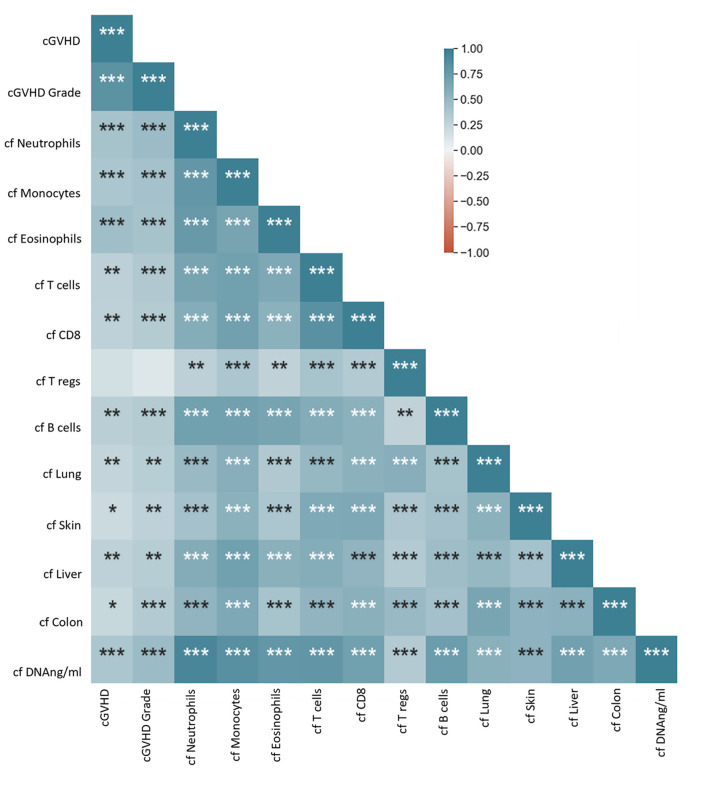
Matrix of correlations between cfDNA and clinical parameters among patients who underwent transplants. Spearman rank correlation coefficient and significance of correlations *P < 0.05; **P < 0.01; ***P < 0.001. cf, cell free DNA; cfDNA ng/mL, total cell free DNA levels in ng/mL.

We next sought to identify correlations between cfDNA parameters and cGVHD clinical scores among patients undergoing HCT. We produced a correlation matrix for all 101 plasma samples for which all tested parameters were available. cfDNA parameters were highly correlated internally — for example, samples with high concentration of total cfDNA tended to also have high levels of organ specific cfDNA (Figure 5 and Supplemental Figure 2) — and there was a significant internal correlation among cGVHD clinical scores, between cGVHD severity assessment and specific organ grading (Supplemental Figures 3 and 4). Moreover, we found a significant correlation between clinical cGVHD severity assessment and total cfDNA as well as organ specific cfDNA levels (Figure 5 and Supplemental Figures 3–5).

### A combined score for blood-based detection of cGVHD.

**Figure 6:**
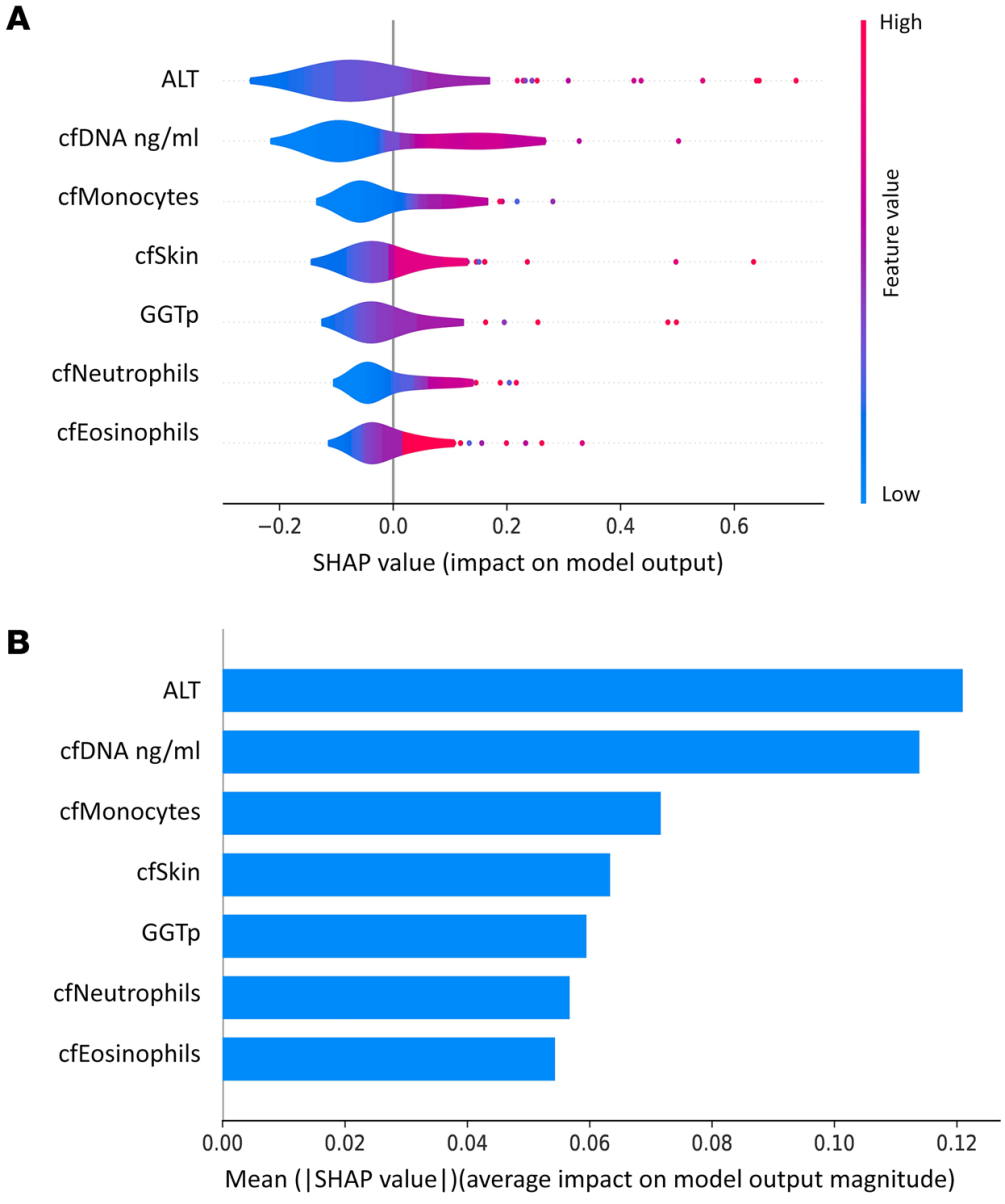
Shapley analysis of cfDNA and clinical features. Evaluation of the contribution of each feature to the model’s prediction of cGVHD. The parameter space of the model was constrained to be nonnegative for all coefficients, thus showing only features with a nonnegligible coefficient. (**A**) Shapley value distributions (**B**) The average absolute SHAP value for each individual feature (ALT, cfDNA ng/mL, cfMonocytes, cfSkin, GGTp, cfNeutrophils, cfEosinophils). cf, cell free DNA; cfDNA ng/mL, total cell free DNA levels in ng/mL; ALT, alanine transaminase; GGTp, γ glutamyl transpeptidase.

**Figure 7:**
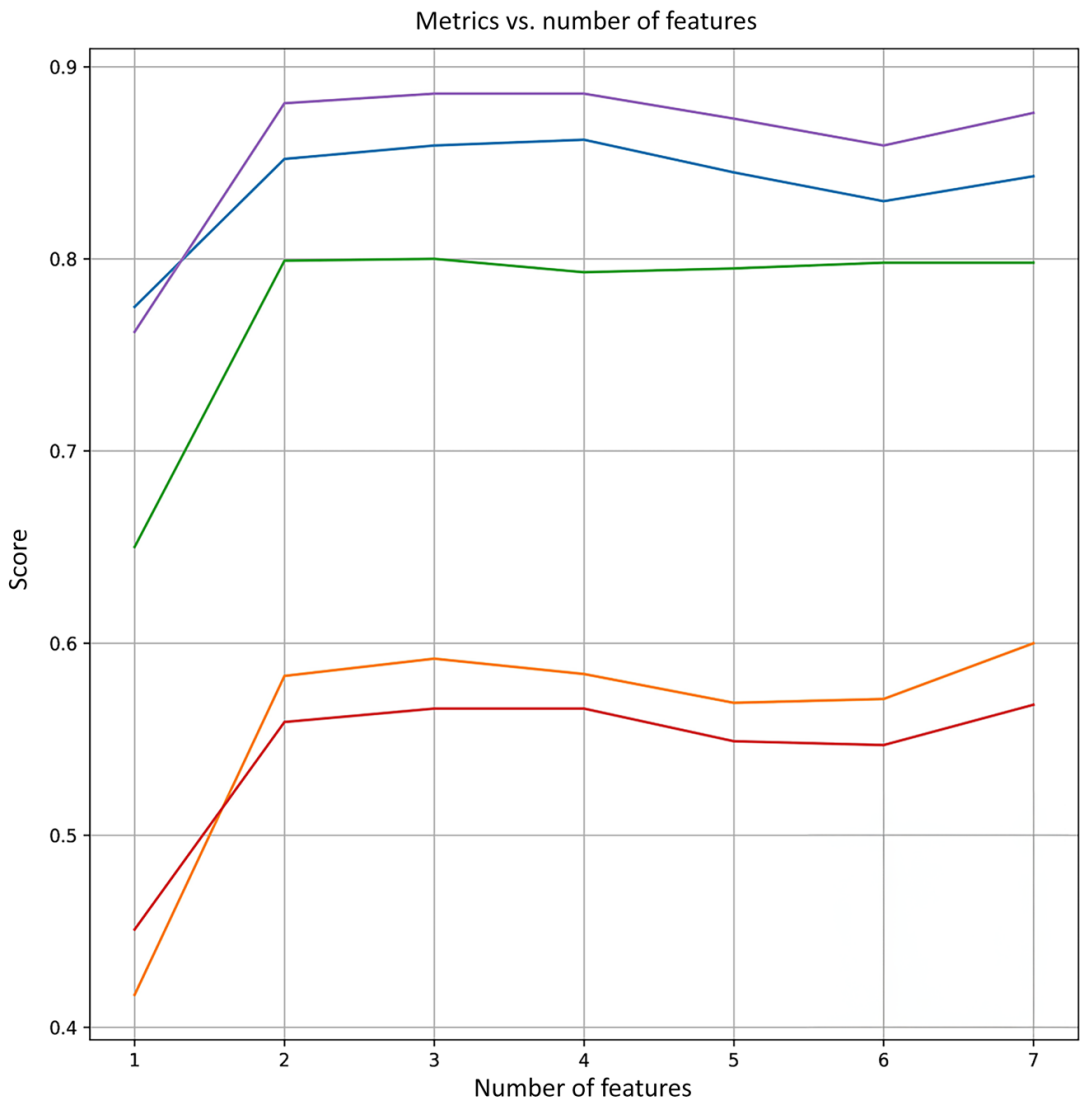
Variation of metrics based on number of features. Visual representation of metrics (specificity, sensitivity, AUC, NPV, and PPV) based on the addition of features according to their importance as determined by Shapley analysis. Purple line, PPV; blue line, specificity; green line, ROC; orange line, sensitivity; red line, NPV.

**Figure 8:**
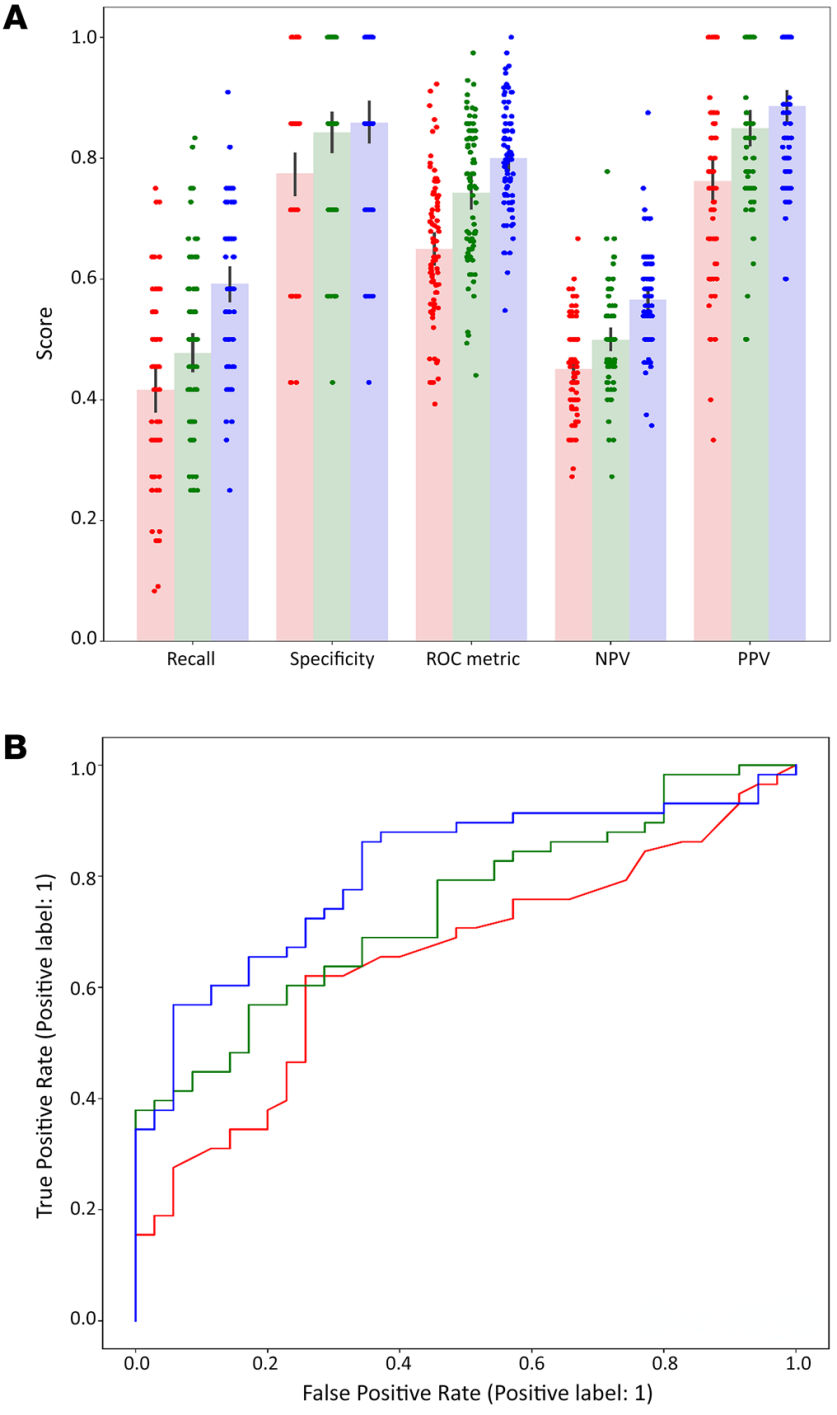
Repeated 5-fold cross-validation results on the best feature set. (**A**) Bar plot of metrics for solely clinical laboratory features (red, ALT), solely cfDNA features (green, cfDNA ng/mL, and cfMonocytes) and combined (blue, ALT, cfDNA ng/mL, and cfMonocytes) (**B**) ROC curves for solely clinical laboratory features (red, ALT; AUC = 0.65), solely cfDNA features (green, cfDNA ng/mL, and cfMonocytes; AUC = 0.74) and combined (blue, ALT, cfDNA ng/mL, and cfMonocytes; AUC = 0.81). cf, cell free DNA; cfDNA ng/mL=cell free total DNA levels in ng/mL.

We wished to create a model that could aid the treating physician to predict the likelihood that a patient has active cGVHD. Employing Shapley analysis (28) on 17 clinical and cfDNA features (see Methods) yielded positive Shapley values for 7 features, including alanine transaminase (ALT), total cfDNA, cfDNA of monocytes, cfDNA of skin, GGTp, cfDNA of neutrophils, and cfDNA of eosinophils. Figure 6A shows distribution graphs for these features, and Figure 6B shows the average absolute Shapley values for each individual feature. We conducted repeated 5-fold cross-validations across these 7 feature sets, starting from the feature having the highest value, ALT, and sequentially adding the next feature in line (e.g., ALT and total cfDNA; ALT, total cfDNA, cfDNA of monocytes, etcetera). The metrics (specificity, negative predictive value [NPV], positive predictive value [PPV], AUC, and precision) for each number of features selected are illustrated in Figure 7. Notably, the 3 first features maximize the AUC, as well as displaying favorable behavior across the other metrics. Therefore, we opt for these 3 features (consisting of ALT, total cfDNA,and cfDNA of monocytes) as the optimal feature set. Recall, specificity, AUC, NPV, and PPV of logistic regression models trained using only ALT, only cfDNA features (total cfDNA and cfDNA of monocytes), and all 3 features are shown in Figure 8A. The ROC curves of these models are shown in Figure 8B.

Finally, we compared the performance of our models to the exact equivalent set of models, where, instead of using a constrained optimization, we used an unconstrained optimization (allowing negative coefficients). Shapley values are shown for all 17 features (Supplemental Figure 6). The metrics (specificity, NPV, PPV, AUC, and precision) for each are illustrated in Supplemental Figure 7. Favorable behavior across all metrics was reached at (ALT, γ glutamyl transpeptidase (GGTp), total cfDNA, cfMonocytes, cfEosinophils, and alkaline phosphatase (ALP)) and repeated 5-fold cross-validation was performed to compare the recall, specificity, AUC, NPV, and PPV of logistic regression models trained using these features (Supplemental Figure 8A), as well as the ROC curves of these models (Supplemental Figure 8B).

Evidently, both constrained and unconstrained optimization techniques demonstrate comparable performance, suggesting minimal overfitting with either optimization technique. Moreover, the findings emphasize the high predictive capability of a small set of features, consisting of biochemical and cfDNA measurements. This aligns with our hypothesis that cGVHD leads to increased cell death, consequently elevating the levels of the observed markers.

## Discussion

Our study shows that tissue-specific DNA methylation patterns can serve as plasma biomarkers for detection of tissue turnover in cGVHD. We demonstrated a general elevation in cfDNA concentration in patients with cGVHD, and an elevation of cfDNA from specific organs as well as immune and inflammatory cells. Combining cfDNA markers with standard biochemical markers allowed us to discriminate patients with and without cGVHD with good sensitivity, accuracy, and precision, suggesting feasibility of a blood-based objective assessment of disease.

In agreement with our findings, it has been demonstrated that mitochondrial cfDNA (COX1 DNA) is higher in patients undergoing SCT compared with people who are in the normal control group and correlates with the presence of cGVHD (29). To our knowledge, this is the first report of potential tissue-specific cfDNA utility in the context of chronic GVHD. Our findings are consistent with, and expand upon, recent studies that focused on the distinct setting of aGVHD (30). Cheng et al. used a smaller number of patients (*n =* 27) and performed shallow whole genome bisulfite sequencing followed by deconvolution, to assess the levels of cfDNA from different recipient sources. Their key finding was that aGVHD — within the first 3 months after HCT — was associated with elevated levels of cfDNA from solid organs (multiple tissues combined). Waterhouse et al. (31) demonstrated substantial differences in the concentration of 1 colon-specific and 1 liver-specific cfDNA marker in 10 and 14 patients with liver and colon aGVHD, respectively. Moreover, they have demonstrated a decline in these markers in patients who were successfully treated. The clinical condition that we studied — cGVHD — is more challenging, as clinical manifestation is typically less abrupt and therefore tissue damage/turnover, which might be reflected by elevated cfDNA levels, is likely to increase gradually. In addition, our approach differs from Cheng et al. in that we use a PCR-Seq of targeted methylation markers to assess the contribution of specific cell types — a method that gives up breadth, i.e., information obtained is limited to a preplanned subset of tissue sources — for specificity, depth, simplicity and low cost. In contrast to the Waterhouse study, our methodology probes multiple indicators for each organ, allowing us to examine a broader spectrum of damaged end organs. Altogether, these 3 studies support the notion that organ damage/turnover and immune deregulation in GVHD are amenable for a cfDNA methylation–based analysis, and that liquid biopsies can be developed into an objective, quantitative, clinically useful tool aiding the treating physician in diagnosing chronic as well as acute GVHD.

An important observation of our study was that most patients undergoing HCT with no clinically detected GVHD had an elevated concentration of the cfDNA derived from donor T cells and recipient intestine, liver, and lung. We propose that this is a reflection of inflammation and increased cellular turnover in patients undergoing HCT, which are taking place even while clinically evident organ function remains in the normal range. This idea is consistent with the model proposed by Cooke et al., whereby cGVHD develops through early inflammation and tissue injury, chronic inflammation, dysregulated immunity, and, eventually, aberrant tissue repair leading to fibrosis (32). Our study was not designed to test if elevated cfDNA from a given tissue source is predictive of future cGVHD. Additionally, larger cohorts will be needed to assess the prognostic potential of methylation-based biomarkers. Such studies will also be able to test the provocative idea that elevated tissue-specific cfDNA takes place chronically even without an overt clinical manifestation, reflecting a low level of allogeneic damage to host tissues that is offset by organ regeneration.

Our study also provides a distinct angle on the nature of immune processes taking place in cGVHD. Extensive studies have revealed the involvement of reactive donor T cells (mainly Th/Tc17), thymic dysfunction, reduced memory B cell formation concomitant with enrichment of alloreactive B cells, reduced levels of T follicular helper cells, macrophage tissue sequestration and activation, and more (3, 16, 17). These studies have typically not characterized immune cell turnover. Our findings suggest that allogeneic HCT causes high turnover of donor T cells (including CD8 and, to a lesser extent, regulatory T cells), at any point after HCT and more so during cGVHD. We hypothesize that the elevated turnover of donor adaptive and innate immune cells, even years after HCT, results from the continuous interaction with allogeneic host tissues. The mechanisms and implications of this phenomenon remain to be elucidated. At a practical level, it is possible that combining cfDNA biomarkers of solid tissues with cfDNA biomarkers of inflammatory / immune cells may increase the specificity of liquid biopsies, e.g. will allow us to differentiate organ damage due to immune attack from damage due to other etiologies (33). Further studies are needed to examine this intriguing possibility.

Implementation of tissue-specific cfDNA biomarkers in clinical GVHD will require additional studies to optimize specificity and sensitivity and to understand how cfDNA dynamics relate to and predict clinical phenotype. We note that the nature of DNA methylation offers a tremendous potential for refining cfDNA analysis. For example, methylation atlases (21, 34) allow us to develop new methylation markers that are specific to hepatocytes from different zones in the liver, alveolar or bronchial epithelial cells, epithelial cells of different segments of the intestine, as well as subsets of immune cells such as tissue-specific macrophages. With regard to sensitivity, emerging lessons from cfDNA-based early cancer detection suggest that parallel assessment of multiple specific markers in the same plasma sample can boost sensitivity by increasing the chance of identifying cfDNA from the tissue of interest. Such refinements of cfDNA assays may facilitate discrimination between suggested biological subgroups of GVHD (resolved GVHD, active late aGVHD, active cGVHD, inactive cGVHD and no GVHD), which is challenging in the current clinical setting. In particular, it will be important to search for cfDNA biomarkers that distinguish active chronic from inactive chronic disease, given the relevance for treatment decisions (32). We note that while our results reveal a good correlation between cfDNA and clinical overt cGVHD, there are outliers showing high cfDNA with no clinical disease and low counts with clinically graded disease. We propose that this discrepancy partly results from the inability of the current clinical grading system to accurately assess the active versus inactive state of cGVHD. Longitudinal studies assessing this hypothesis are warranted.

Using multivariate logistic regression, we pinpointed a trio of pivotal features (ALT, total cfDNA concentration, and monocyte/macrophage cfDNA concentration) yielding compelling results: specificity of 86%, a positive predictive value (PPV) of 89%, and a robust AUC value of 0.8. Notably, what we believe sets our approach apart is its efficiency in capitalizing on a balanced selection of a single biochemical parameter and a pair of distinct cfDNA parameters. This pragmatic strategy not only streamlines the diagnostic process but also markedly enhances the ability to accurately discern cases of cGVHD. Conceptually, we believe that combined measurements of classical markers such as liver enzymes and cell counts with cfDNA biomarkers is expected to synergize. The reason is that cfDNA provides distinct biological information; it reveals cell turnover, which is different from cell counts; it is a definitive marker of cell death (while cytoplasmic proteins may be released to blood upon transient cell injury); and it is cleared rapidly, revealing information about acute tissue damage. Particularly intriguing is the role of cfDNA derived from monocytes and macrophages, suggesting a pivotal role of macrophage turnover in the context of cGVHD. This observation is consistent with current understanding of the contribution of macrophage infiltration and activation within affected organs to the pathobiology of the disease (35).

Our study has several limitations. First, we acknowledge that the assay used in this study, based on massively parallel sequencing, may pose challenges to implementation in a standard clinical setting. However, as we have shown before, the small number of target loci makes it possible for translation into a simpler version, based on quantitative PCR (20). Such a version will have the advantage of delivering results faster (same day), at a low cost and in a point-of-case setting. Second, the process underlying elevated tissue-specific cfDNA is not fully understood; for example, it could reflect an increased rate of cell death in the tissue of origin, enhanced turnover rate, or disruption of local removal of debris from dying cells. Regardless, elevated cfDNA appears to correlate well with clinical cGVHD. Third, our study was designed for diagnostic purposes only and not for predictive, prognostic, or response to treatment purposes. These need to be explored in separate, well-designed, prospective, longitudinal studies including a large cohort of patients who have undergone transplants. This research should delve into the changes in cfDNA patterns over various time points, commencing before the conditioning protocol, spanning the transplantation phase, and encompassing the periods of acute and chronic GVHD. Fourth, this is a single center study and further validation studies using independent cohorts from additional centers are needed.

In conclusion, we demonstrate the potential utility of tissue-specific methylation markers for objective and clinically useful detection of cGVHD. We envision that cfDNA biomarkers can transform GVHD treatment into a highly personalized process, where patients are monitored by liquid biopsy many times after transplant and during treatment to monitor disease and adjust treatment.

## Methods

### Methylation analysis.

We prepared cfDNA and measured its concentration (in nanograms per milliliter plasma), then treated with bisulfite to expose the status of methylation and performed multiplex PCR as described (36) to amplify marker loci. PCR products were sequenced on a NextSeq machine (Illumina), and the fraction of molecules carrying a tissue-specific pattern of methylation was determined. We used this information, averaged over the markers for each tissue, to assess the relative contribution of each tissue to cfDNA. In addition, by multiplying the proportion of cfDNA from each tissue by the total concentration of cfDNA in a sample, we calculated the absolute concentration of cfDNA from each tissue to plasma, expressed in genome equivalents per milliliter plasma, as described (27, 36). Primer sequences of all markers, as well as clinical and methylation data for all samples are provided in Supplemental Tables 1 and 2.

### Clinical assessment of patients undergoing HCT in the chronic setting.

To assess the utility of cfDNA for detecting organ damage in cGVHD, we prospectively collected 101 plasma samples from 101 individuals more than 100 days after allogeneic stem cell transplantation, arriving for planned routine clinical follow up, at the Bone Marrow Transplant (BMT) day care unit at Hadassah medical center. Upon each visit blood was drawn for regular blood tests (extra 10 mL of blood was drawn for cfDNA analysis) and the patient underwent a full assessment by the treating physician which included cGVHD grading according to the 2014 NIH criteria. During the course of this 38-month study, 65 patients were diagnosed at any point with clinically evident cGVHD, while 36 were not found to have clinical signs of cGVHD.

### Patient characteristics.

The median age of patients was 47 years. A total of 65% of the patients were men and 35% were women. The majority of patients underwent transplant due to acute myeloid leukemia (57%), had a matched sibling (63%), were treated with a myeloablative conditioning regimen (64%) and received stem cells withdrawn from peripheral blood (PBSC, 92%). Most of the patients (55%) received a transplant from a matched sex, while 25% were transplanted from a mismatched donor sex, in a female to male direction.

Of the 101 samples, 57% were collected from patients with a history of aGVHD. None had signs of overlap (both acute and chronic) GVHD at the time of sampling. A single patient developed liver GVHD 1 month after Donor Lymphocyte Infusion (DLI). The median time from transplantation was 783 days (range 101–7,878 days). Half of the samples (*n* = 49 samples) were taken from patients receiving 1 or more immunosuppressive agents at the time of collection. Only 7 patients had evidence of CMV viremia at the time of collection. One patient had biopsy-proven colitis, which did not show CMV inclusion bodies, while none of the remaining 6 had any evidence for CMV disease. Four patients were treated for CMV infection. Eleven patients had a positive EBV-PCR in peripheral blood (with a median of 300 copies/mL), none of which was clinically significant. One patient was positive in the upper respiratory tract for RSV and another for influenza. One patient had staphylococcus epidermis bacteremia. Chimerism levels were routinely monitored. 98% of the samples were obtained from patients with a blood driven STR assay indicating 100% donor-derived hematopoietic cells. Two samples exhibited a donor chimerism ranging from 88%–92%, precluding analysis of the relationship between degree of chimerism, cfDNA methylation profiles, and a potential relapse. None of the samples were taken at the time of relapse.

### Statistics.

Assessment of cfDNA plasma levels in healthy controls versus allogeneic transplanted patients with and without clinical signs of cGVHD was performed using nonparametric, unpaired, Mann Whitney test. Analyses were performed using GraphPad Prism (version 10.0.1), and results were considered statistically significant for *P* values of less than 0.05.

We used machine learning to evaluate the predictive power of both cfDNA and biochemical measurements in relation to clinical evident cGVHD. We compared multivariate logistic regression (MLR), XG boost and random forest (RF) classifiers on our data set. MLR, XGboost, RF had an average accuracy of 0.74, 0.67, and 0.65, respectively, by Repeated-K-fold cross-validation (K = 5) with a SD of 0.23, 0.22, and 0.3, respectively. As the MLR model had both higher accuracy with similarly robust results by cross validation, we applied MLR for further analyses. Furthermore, MLR emerges as the most fitting estimator based on the following considerations: (a) We anticipate that GVHD will consistently increase the levels of measured markers, signifying increased cell death. Hence, a monotonous model that consistently increases in response to changes in its features should be appropriate. (b) The size of our data does not support models with a large number of parameters — MLR bears a single parameter per measurement, reducing the risk of overfitting. (c) MLR inference naturally provides a probability score.

We hypothesize that measurements of cfDNA and blood biochemical values possess significant predictive potential for the presence of cGVHD. We leveraged Shapley values to gauge the magnitude of the predictive capability of each feature. This latter technique offers a principled approach to feature selection, promoting enhanced performance with reduced overfitting. Since we expect higher cfDNA levels to indicate cGVHD, we constrained the parameter space of the model to be nonnegative for all coefficients and compared the performance to an unconstrained optimization in order to explore the overfitting potential of the model. A total of 93 samples (for which data was available for all parameters) were used for the analysis. We employed Shapley analysis (28) on a collection of 17 features (comprising of GGT, ALP, ALT, AST, TBil, Total cfDNA level [presented in ng/mL], and organ specific cfDNA: cfSkin, cfLung, cfGI, cfLiver, cfNeutrophils, cfMonocytes, cfEosinophils, cfB cells, cfT cells, cfCD8 cells, and cfTregs cell). Next, to robustly validate the predictive potential of the features, we utilized Repeated-K-Fold cross-validation (37). We conducted repeated 5-fold cross-validations across the feature sets given a positive coefficient (constrained optimization). Each set, labeled, consists of the highest-ranking features, meaning, set =1 is the single top-ranking feature, set =2 consists of the 2 top ranking features, set =3 of the 3 top ranking features and so on. The metrics (Specificity, NPV, PPV, AUC, and Precision) for each were calculated. The selection of the best feature set was determined based on those achieving the highest AUC and demonstrating favorable performance across other metrics. We calculated recall, specificity, AUC, NPV, and PPV of logistic regression models trained using only the best feature set. A comparison of cfDNA features compared with blood biochemical features and with the combination of both (meaning the entire set) was performed. All analyses were performed using Python 3.10.

Using the model, accuracy ([(TP+TN)/Total testing samples] x100%), specificity ([TP/(TP+FN)] x100%), sensitivity([TN/(TN+FP)] x100%), PPV ([TN/(TN+FN)] x100%) and NPV/precision ([TP/(TP+FP)] x100%) were measured.

Graphical representation of the tradeoff between specificity and sensitivity was done using the receiver operating characteristics curve (ROC). AUC was calculated in order to determine the ability of the classifier to distinguish positive and negative results. Spearman rank correlation was used to determine the significance of correlation between each pair of variables and other parameters.

### Study approval.

The study was approved by the Hadassah Medical Center IRB committee and is consistent with the declaration of principles of Helsinki. Written informed consent was received prior to participation.

### Data availability.

Primer sequences of methylation markers, as well as clinical and methylation data values for all samples are provided in Supplemental Tables 1 and 2. Values for all data points in graphs can be found in the Supplemental Table 5: Supporting Data Values file.

## Author contributions

BA, BG, SJS, RS, and YD designed the research study. BA, DN, OGR, SG, IFF, and SP conducted experiments. BA, ES, OGR, IA, BG, SJS, RS, YD, and JM analyzed data. BA, SG, MK, AF, PS, TZ, and ALS provided reagents. BA, ES, IA, BG, RS, YD, and JM wrote the manuscript. BA and DN are co–first authors. The order of names in the paper, as well as the order of co–first authors, was determined based on the relative contributions, which were in different areas (study design, patient access and data interpretation, methylation analysis, and statistical analysis).

## Supplementary Material

Supplemental data

ICMJE disclosure forms

Supplemental table 1

Supplemental table 2

Supporting data values

## Version 1 11/16/2023

In-Press Preview

## Version 2 01/16/2024

Electronic publication
