# Supplementary material for: Chronic graft-versus-host disease detected by tissue-specific cell-free DNA methylation biomarkers
Source: J Clin Invest. 2024 Jan 16;134(2):e163541. doi: 10.1172/JCI163541 (PMC10786696; doi:10.1172/JCI163541)

Supplementary materials

Table legends:

Supplementary Table S1: attached EXCEL file

Marker coordinates and primer sequences.

Supplementary Table S2: attached EXCEL file

Clinical and methylation data for all samples.

Supplementary Table S3: Patient characteristics.

Myeloablative, Reduced intensity and non-myeloablative are the following (based on CIBMTR consensus published in BBMT 2009): **Myeloablative:** TBI ≥ 500 cGy as a single fraction or ≥ 800 cGy if fractionated, Total busulfan ≥ 9 mg/kg, Total melphalan ≥ 150 mg/m^2, Thiotepa ≥ 10 mg/kg. **Non-Myeloablative:** Busulfan ≤ 6.4 mg/kg. **Reduced Intensity Conditioning:** Regimens that do not meet the criteria for MA and NMA.

***Abbreviations:** AML- acute myeloid leukemia; ALL-acute lymphoid leukemia, MSD-matched sibling donor; MRD-matched related donor; MUD-matched unrelated donor; Haplo-haploidentical donor; PBMC-peripheral blood stem cells; BM-bone marrow; MA-myeloablative; RIC-Reduced intensity, NMA-nonmyeloablative; UK-unknown; CSA-cyclosporine A; MTX-methotrexate; MMF-mycophenolate mofetil; PTCY=posttransplant cyclophosphamide; TAC=Tacrolimus; M=Male; F=Female

Supplementary Table S4: post transplantation patients characteristics.

***Abbreviations: UK=unknown; CSA=cyclosporine; MTX=Methotrexate; ECP=extracorporeal photopheresis; GI-=Gastrointestinal.

Supplementary Table S5: Supporting Data Values file.

Supplementary Figure legends:

Supplementary Figure S1: Tissue specific cell free (cf) DNA levels correlate with clinical tissue chronic GVHD.

Tissue-specific cfDNA in healthy volunteers and in allogeneic HCT patients in relation to clinical chronic GVHD score in skin (A), lung (B) and liver(C). Each dot represents one plasma sample.

Supplementary Figure S2: Matrix of correlations between cfDNA and clinical parameters among transplanted patients. Spearman's rank correlation coefficient is listed for each comparison.

*Abbreviations: cf=cell free DNA; cfDNAng/ml=Total cell free DNA levels in ng/ml; cGVHD=chronic Graft Versus Host Disease.

Supplementary Figure S3: Correlation matrix between cfDNA and specific organ cGVHD scoring among transplanted patients. Spearman's rank correlation coefficient and significance of correlations (*<0.05 **<0.01 ***<0.001).

*Abbreviations: cf=cell free DNA; cfDNAng/ml= Total cell free DNA levels in ng/ml; cGVHD=chronic Graft Versus Host Disease; cOrgan=clinical organ involvement with cGVHD.

Supplementary Figure S4: Correlation matrix between cfDNA and specific organ cGVHD scoring among transplanted patients. Spearman's rank correlation coefficient listed for each comparison.

*Abbreviations: cf=cell free DNA; cfDNAng/ml= Total cell free DNA levels in ng/ml; cGVHD=chronic Graft Versus Host Disease; cOrgan=clinical organ involvement with cGVHD.

Supplementary Figure S5: Liver cfDNA levels correlate with liver enzyme levels.

Liver enzyme levels: ALT, AST, ALP, GGTp- represented in units/L and TBIL levels in micromole/L. Each dot represents one plasma sample.

*Abbreviations: ALT- Alanine transaminase, AST- Aspartate transaminase; ALP- Alkaline phosphatase; GGTp- gamma glutamyl transpeptidase; TBIL- Total bilirubin.

Supplementary Figure S6: Shapley analysis of cfDNA and clinical features. Evaluation of the contribution of each feature to the model's prediction of cGVHD. The parameter space of the model was non constrained thus showing all 17 measured features. A**.** SHAP value distributions **B.** The average absolute SHAP value for each individual feature (GGTp, ALP, ALT, AST, TBil, total cfDNA level, cfSkin, cfLung, cfGI, cfLiver, cfNeutrophils, cfMonocytes, cfEosinophils, cfB cells, cfT cells, cfCD8 cells, cfTreg cells). *Abbreviations: cf=cell free; cfDNAng/ml= Total cell free DNA levels in ng/ml;

Supplementary Figure S7: Variation of Metrics based on Number of Features. Visual representation of metrics (specificity, sensitivity, AUC, NPV, PPV) based on the addition of all 17 features according to their importance as determined by SHAP analysis. (purple line=PPV, blue line=specificity, green line=ROC, orange line=sensitivity, red line=NPV).

*Abbreviations: NPV-Negative Predictive Value, PPV-Positive Predictive Value.

Supplementary Figure S8: repeated 5-fold cross validation results on the best feature set.

A. Bar plot of metrics for solely clinical laboratory features (red; ALT, GGTp, ALP), solely cfDNA features (green; cfDNAng/ml, cfMonocytes, cfEosinophils, cfNeutrophils) and combined (blue; ALT, GGTp, ALP, cfDNAng/ml, cfMonocytes, cfEosinophils, cfNeutrophils) B. ROC curves for solely clinical laboratory features(red; ALT, GGTp, ALP; AUC=0.68), solely cfDNA

features (green; cfDNAng/ml, cfMonocytes, cfEosinophils, cfNeutrophils; AUC=0.74) and combined (blue; ALT, GGTp, ALP, cfDNAng/ml, cfMonocytes, cfEosinophils, cfNeutrophils; AUC=0.80).

*Abbreviations: cf=cell free DNA.

Supplementary Table S3: Sample characteristics.

Variables	Number of Patients (Total=101) (%)
**Age (Years)**	
Median (Range)	47(18-74)
**Gender**	
Male	65(64.4%)
Female	36(35.6%)
**Hematological Disease**	
AML	57(56.4%)
ALL	18(17.9%)
LYMPHOMA	11(10.9%)
MDS	7(6.9%)
OTHER	8(7.9%)
**Donor Type**	
MSD	64(63.4%)
MRD	4(3.9%)
MUD	28(27.8%)
HAPLO	4(3.9%)
CORD BLOOD	1(1%)
**Matching**	
Match	77(76.3%)
Mismatch	19(18.8%)
UK	5(4.9%)
**Stem Cells Source**	
PBSC	93(92.1%)
BM	7(6.9%)
UK	1(1%)
**Conditioning Intensity**	
MA	65(64.4%)
RIC	16(15.8%)
NMA	19(18.8%)
UK	1(1%)
**GVHD Prophylaxis**	
CSA	16(15.8%)
CSA+MTX	18(17.9%)
CSA+MMF	61(60.4%)
PTCY+TAC+MMF	5(4.9%)
UK	1(1%)
**Gender Match**	
M to M	45(44.6%)
F to F	11(10.9%)
M to F	19(18.7%)

F to M	25(24.8%)
UK	1(1%)
**ATG**	
Yes	51(50.5%)
No	49(48.5%)
UK	1(1%)

Supplementary Table S4: post transplantation patients characteristics.

Variables	Number Of Samples (Total=101) (%)
**Acute GVHD**	
No	42(41.6%)
Yes	58(57.4%)
UK	1(1%)
**Chronic GVHD**	
No	36(35.6%)
Yes	65(64.3%)
**Chronic GVHD Grade**	**(Total=65) (%)**
Mild	27(41.5%)
Moderate	22(33.9%)
Severe	16(24.6%)
**Prednisone >10MG**	**(Total=101) (%)**
No	86(85.1%)
Yes	15(14.9%)
**Immunosuppressive Agent at time of sampling**	**(Total=47) (%)**
CSA	24(51.1%)
Tacrolimus	18(38.3%)
Sirolimus	3(6.4%)
Ruxolitinib	6(12.8%)
MTX	1(2.1%)
ECP (2 weeks prior)	2(4.3%)
**Chronic Organ Involvement**	**(Total=65) (%)**
Skin	29(44.6%)
Sclerodermatous	14(21.5%)
Lung	22(33.8%)
Liver	12(18.5%)
GI	2(3.1%)
Eyes	38(58.5%)
Mouth	23(35.4%)
Genital	7(10.8%)
Joints And Fascia	12(18.5%)

Supplementary Figures

Supplementary Figure S1: **: Tissue specific cell free (cf) DNA levels correlate with clinical tissue chronic GVHD.**

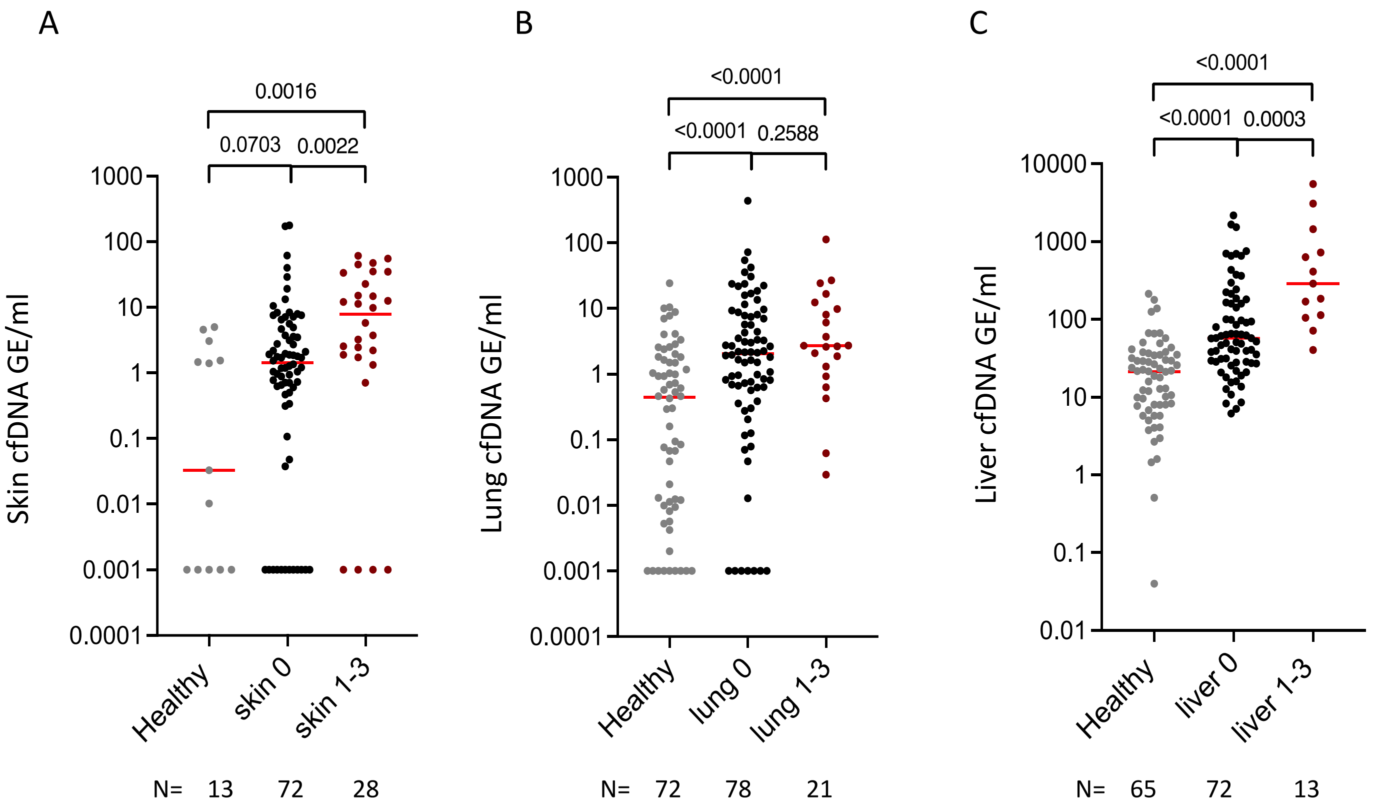

Supplementary Figure S2: Matrix of correlations between cfDNA and clinical parameters among transplanted patients.

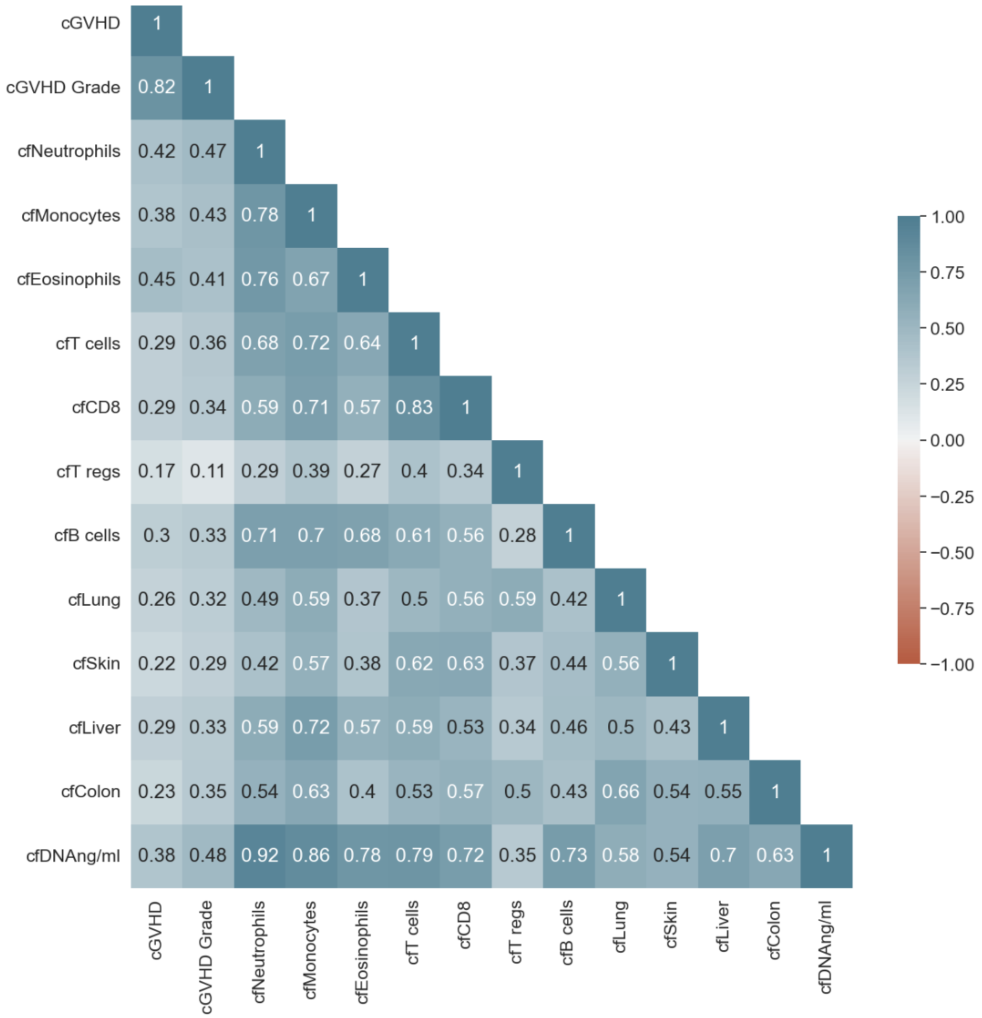

Supplementary Figure S3: Correlation matrix between cfDNA and specific organ cGVHD scoring among transplanted patients.

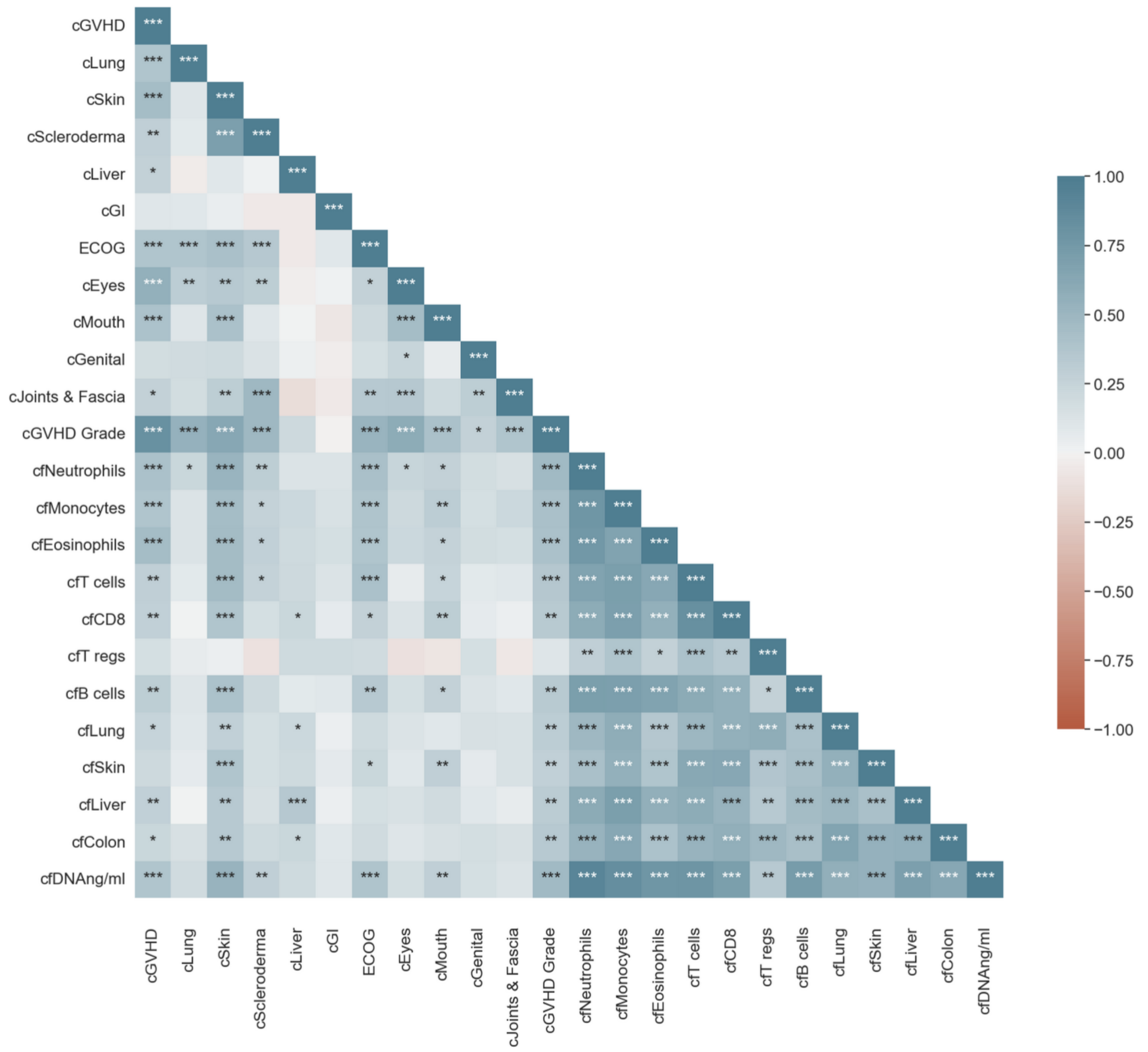

Supplementary Figure S4: Correlation matrix between cfDNA and specific organ cGVHD scoring among transplanted patients.

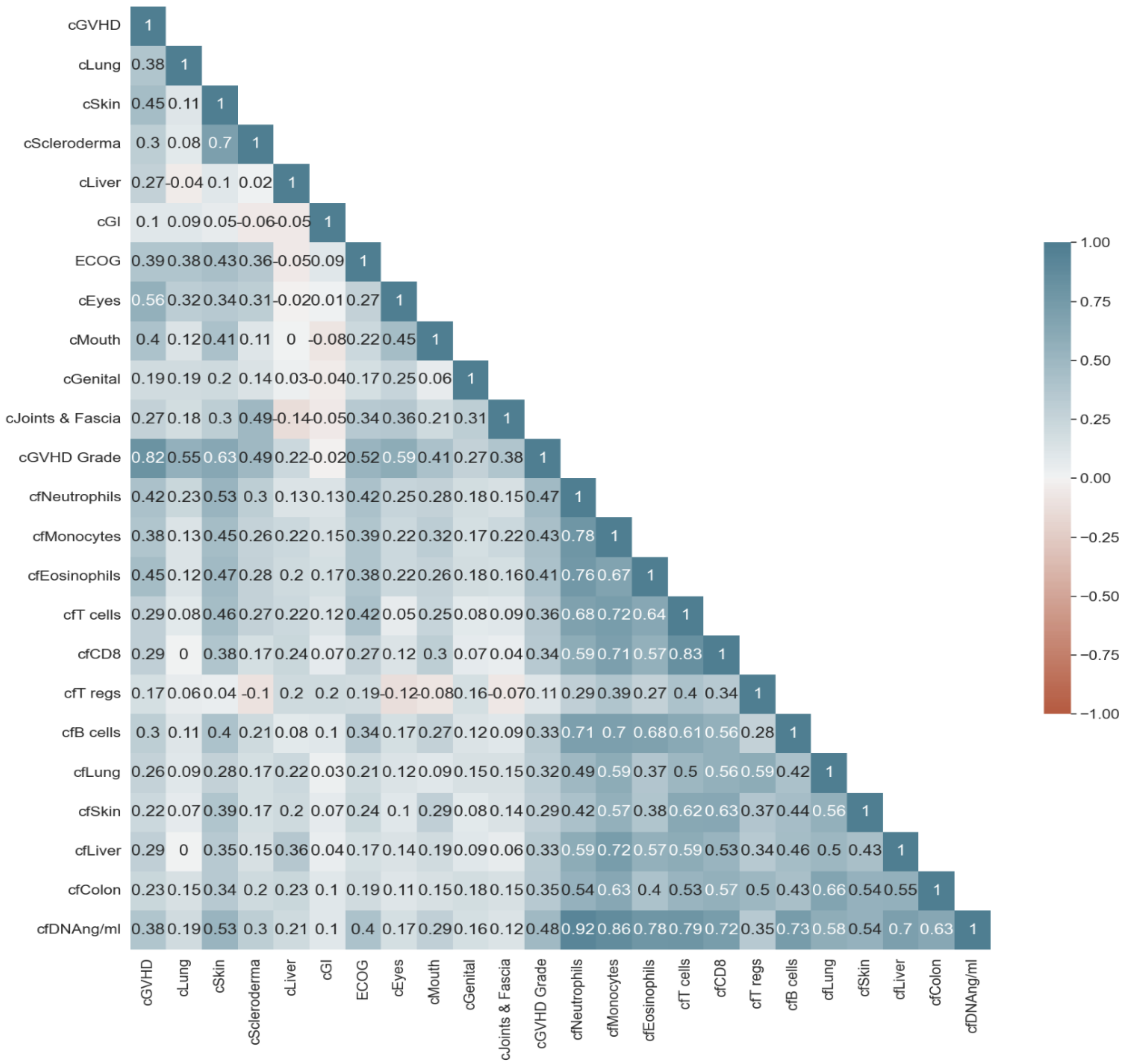

Supplementary Figure S5: Liver cfDNA levels correlate with liver enzyme levels.

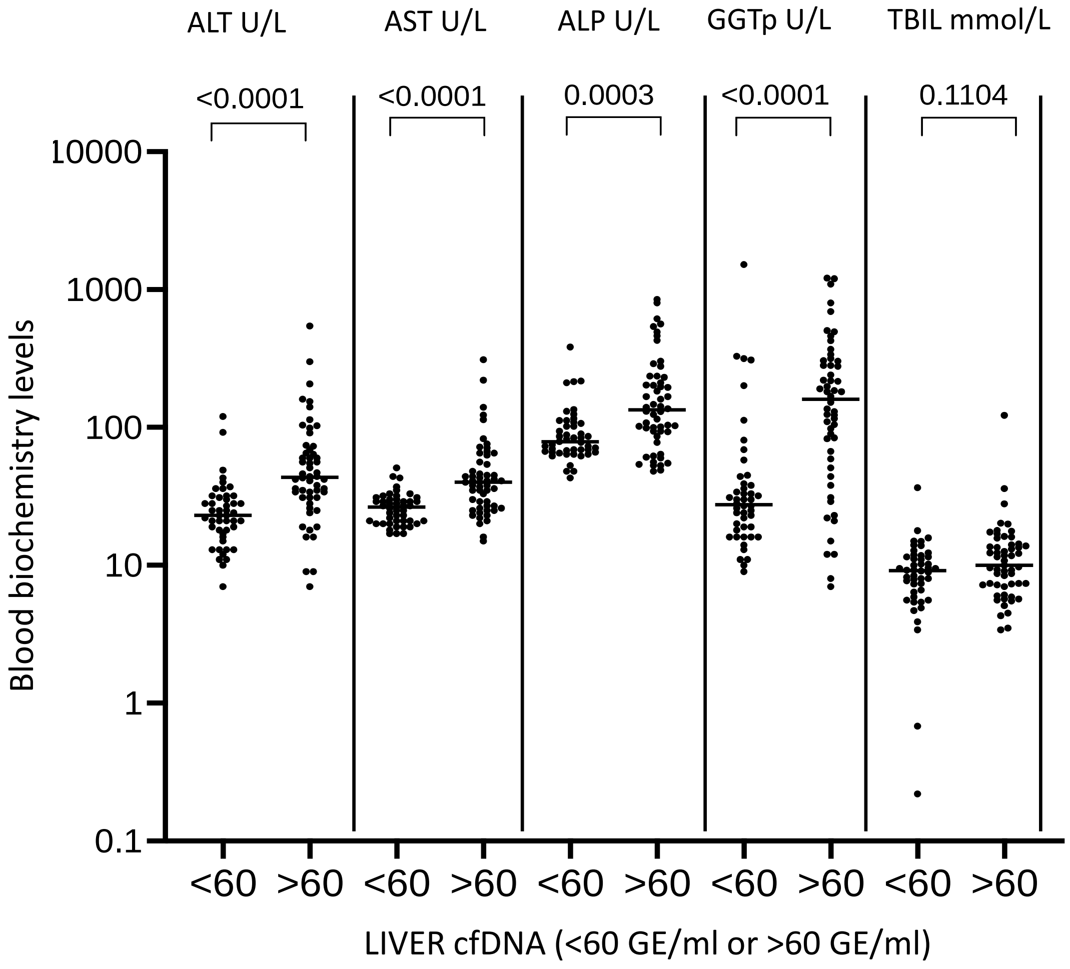

Supplementary Figure S6: Shapley analysis of cfDNA and clinical features.

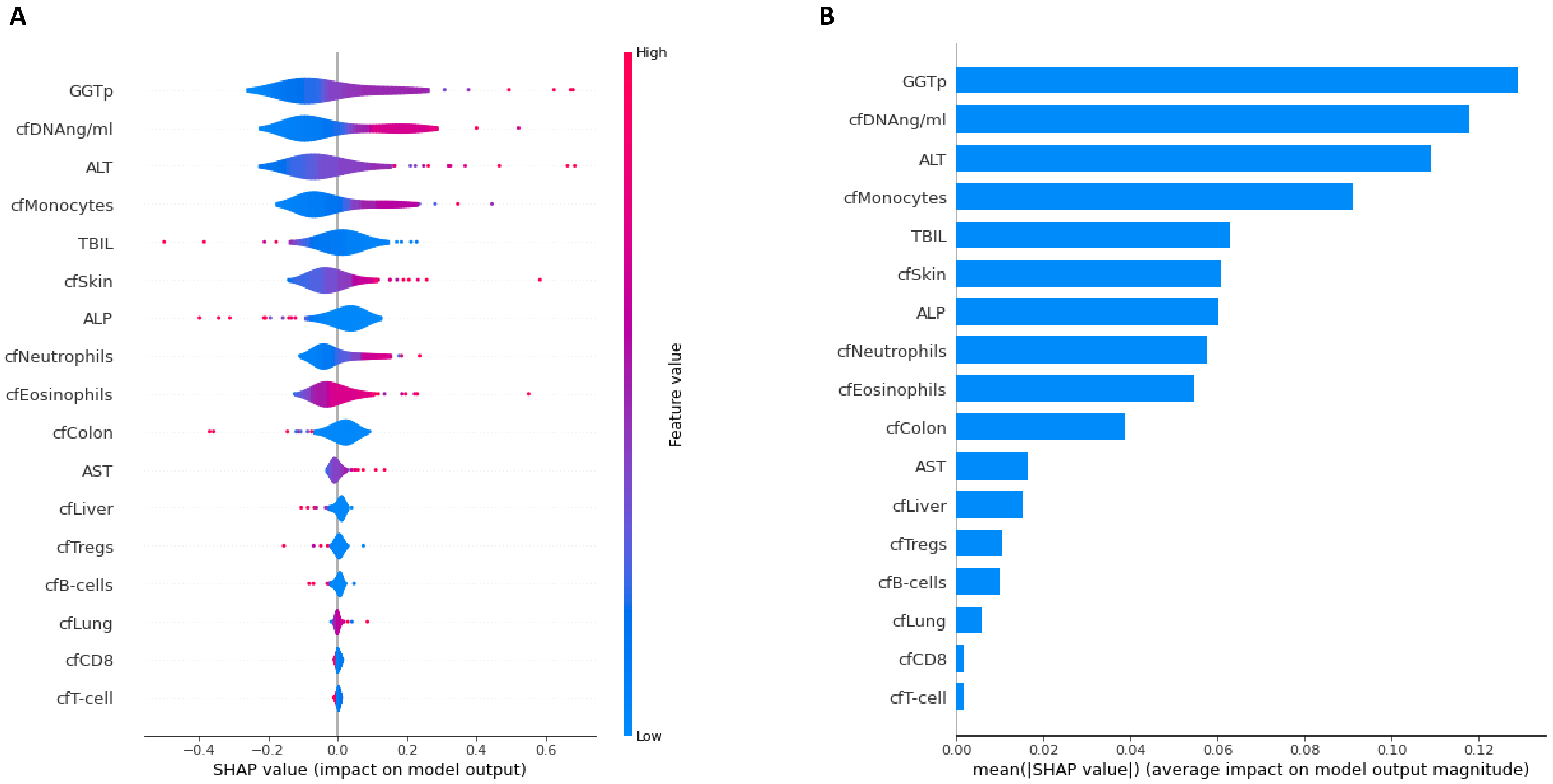

Supplementary Figure S7: **Variation of Metrics based on Number of Features.**

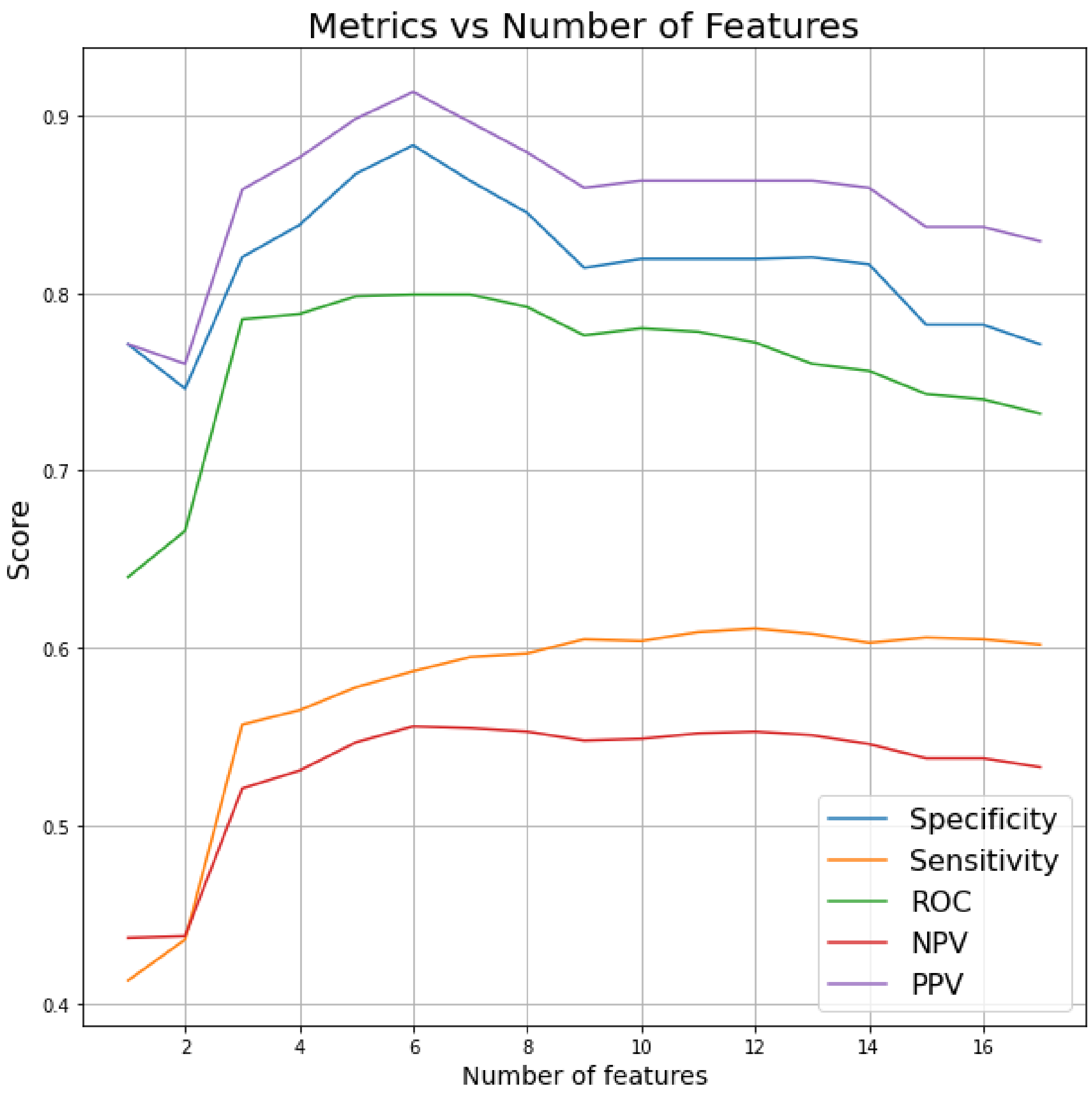

Supplementary Figure S8: Repeated 5-fold cross validation results.

A

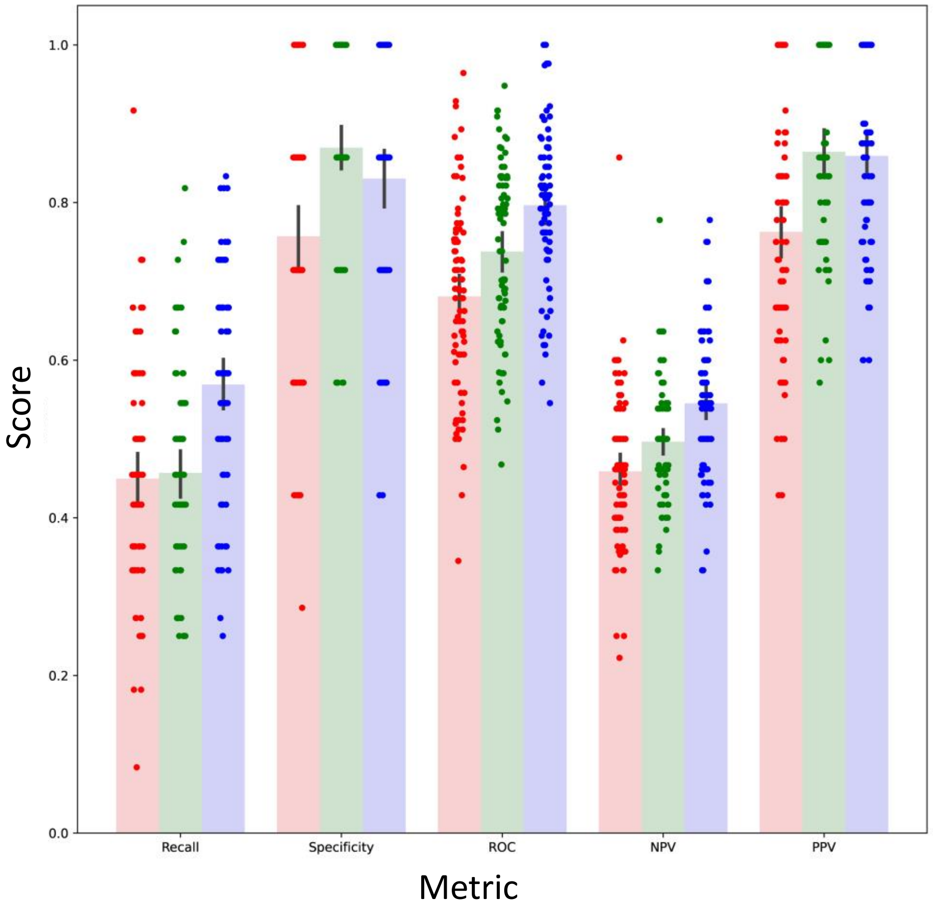

B

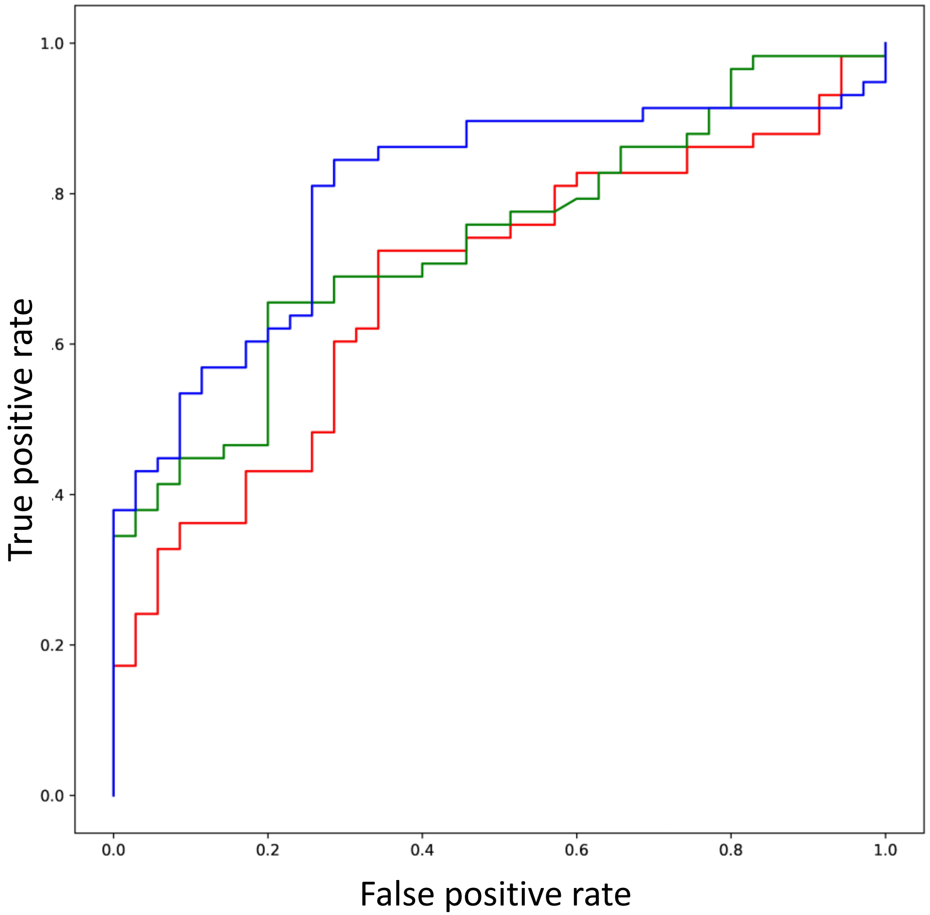

Supplement: Supplemental data [file jci-134-163541-s175.pdf]
